# Complaints: By a Hospital Chairman

**Published:** 1912-04-13

**Authors:** 


### COMPLAINTS: By A HOSPITAL CHAIRMAN.*

### III.—Some Recurrent Difficulties.

I remember one instance where my most sincere sympathies were certainly aroused by the man with a grievance, and yet there were points about the case which might lead one to believe that such cases might occur in any institution.

A man wrote complaining that his wife had taken his little daughter to the hospital suffering from a broken leg, and that owing to infection in the children's ward the house surgeon refused to admit the child and did not in fact treat her at all.

I called by appointment and found the man to be a labouring man, with two children, living in a half-house. He stated that he earned about 21s. per week. The room was scrupulously clean and nicely furnished; the wife, the children, and himself so dressed as to make one prone to believe that they were fairly well to do. The facts, as explained, were interesting. The child fell and broke her leg. The mother took her at once to a doctor close at hand. The doctor had no splint handy so sent her with a verbal message to the hospital. Anyone of experience knows those verbal messages. The woman was, she admitted, greatly agitated, excited, and upset; she was not in good health. How she gave the message may perhaps be imagined. The house surgeon seems to have thought that she could afford a doctor. He probably thought, as a surgeon might, that a green-stick fracture was a matter of very slight importance; and being unable to admit the child, rather casually, I fear, told her to take it back to the doctor who sent her. He stated that had he known of her poverty, or her condition, he would not have done as he did; but he was busy and probably annoyed at the manner or the matter of the message as it reached him.

The poor woman was only awaiting further developments before being admitted to another hospital to have a serious operation performed. She doubtless thought a broken leg a terrible tragedy and every moment that it remained untreated was purgatory to her. They were saving to pay the expenses of the expected event and for a woman to look after the children during her stay in hospital, and therefore did not want to incur a doctor's bill. The man was a total abstainer and all his love and ambition were centred round his home, which means, among his class, wife, family, rooms, and furniture. He readily forgave the hospital authorities, and I had much pleasure in being able to help a most deserving and interesting family in their time of stress.

I was once in the hall when a boy was handed back to his mother "discharged cured." He had met with a very serious accident, and, if I remember rightly, been "in" for eight or nine weeks, then sent to a convalescent home for three. When received his chance of life was well-nigh hopeless. I can see and hear now the scornful way with which his "fond" parent received him! Looking him up and down she said: "Corl this a 'orspital! Why, they ain't even giv' 'im a noo soot orf cloes!"

Recently you criticised a statement of the secretary of an institution which had been the subject of a serious complaint. The secretary stated that there were three patients in the surgical wards more than there were beds for. You say, "And this, again, is an episode on which we cannot congratulate him, or his board."

This raises another question. Is either the secretary or his board responsible for admissions? In my first letter on this subject I pointed out the curious attitude of coroners and their juries with regard to treatment. I remember a similar case to the one mentioned above, where in an institution all the beds were full. I dare not admit that there were more patients than accommodation. The house surgeon, after careful detention and first aid, sent the patient with all precautions to another institution where, after several (I think five) months, the case died—not of the injury but of pneumonia. The coroner and jury blamed "the hospital authorities" of the first institution for "Inhuman treatment," and in similar phrases.

For nearly twenty years I have closely watched the work of one voluntary institution and made myself interested in that of many. For ten years past I have assisted personally in carefully investigating every case of complaint with regard to treatment at the former institution that has come to my notice. I have attended at coroners' courts; sat in the waiting-rooms of various popular doctors; attended meetings of working men and heard and answered their allegations; held inquiries at the institution and at patients' homes at all hours of day and night; and I unhesitatingly say that I have never come across a case that might be described as "inhuman treatment." The case cited at the beginning of this letter is perhaps the nearest to deserving such description of any I remember.

By far the greater number of cases of complaint have been due to the same old reason—no vacant beds. Perhaps some other "authorities" will give their experiences and tell one how the surgeons should act when a serious accident is brought in and *all* beds, medical as well as surgical, are occupied.

To my mind there is only one solution, and that is—more beds. But this involves the expenditure of more money. So let complainants see that they and their friends have all done their duty to provide beds before stoning the "authorities" who, at least, are voluntarily giving time, in some cases much time, and money in attempting to treat freely and as efficiently as possible those wayfarers who, through accident or disease, need the samaritan help so freely offered by so many splendid institutions with the aid of a fine body of voluntary and paid helpers who rejoice in their noble efforts, though so often hampered by chronic lack of space and beds, and last, but not least, "the sinews of war."

Chairman.

* Previous articles in this series appeared in The Hospital of February 24 and March 9.

